# Differential transcription factor expression by human epithelial cells of buccal and urothelial derivation

**DOI:** 10.1016/j.yexcr.2018.05.031

**Published:** 2018-08-15

**Authors:** Arianna Hustler, Ian Eardley, Jennifer Hinley, Joanna Pearson, Felix Wezel, Francois Radvanyi, Simon C. Baker, Jennifer Southgate

**Affiliations:** a Jack Birch Unit for Molecular Carcinogenesis, Department of Biology, University of York, York YO10 5DD, United Kingdom; b Pyrah Department of Urology, St. James's University Hospital, Leeds LS9 7TF, United Kingdom; c Oncologie Moléculaire, Institut Curie, Centre de Recherche, 75248 Paris cedex 05, France

**Keywords:** Urothelium, Buccal epithelium, Transdifferentiation, Nuclear receptor, Transcription factor

## Abstract

Identification of transcription factors expressed by differentiated cells is informative not only of tissue-specific pathways, but to help identify master regulators for cellular reprogramming. If applied, such an approach could generate healthy autologous tissue-specific cells for clinical use where cells from the homologous tissue are unavailable due to disease. Normal human epithelial cells of buccal and urothelial derivation maintained in identical culture conditions that lacked significant instructive or permissive signaling cues were found to display inherent similarities and differences of phenotype. Investigation of transcription factors implicated in driving urothelial-type differentiation revealed buccal epithelial cells to have minimal or absent expression of *PPARG*, *GATA3* and *FOXA1* genes. Retroviral overexpression of protein coding sequences for GATA3 or PPARy1 in buccal epithelial cells resulted in nuclear immunolocalisation of the respective proteins, with both transductions also inducing expression of the urothelial differentiation-associated claudin 3 tight junction protein. PPARG1 overexpression alone entrained expression of nuclear FOXA1 and GATA3 proteins, providing objective evidence of its upstream positioning in a transcription factor network and identifying it as a candidate factor for urothelial-type transdifferentiation or reprogramming.

## 1 Introduction

The urinary tract from the renal pelvis, through ureters, bladder and proximal urethra is lined luminally by urothelium: a transitional epithelium. Urothelium derives from two embryological sources: bladder urothelium is endodermally-derived from the urinogenital sinus, whereas ureteric urothelium is of mesodermal (Wolffian duct) origin [37]. Both urothelia share common features of form and function, with stratification into basal, intermediate and the highly-specialised lumen-facing superficial cells that constitute the main urinary barrier. Superficial cells are characterised by apical expression of tissue-restricted transmembrane uroplakins that contribute to transcellular barrier function [10], [36], whilst paracellular barrier function is provided by well-developed intercellular tight junctions [16], [44]. In human urothelium, expression of claudin 3 is implicated functionally in the terminal tight junction [32]. Not only does urothelium form one of the tightest barriers in the body, but despite being mitotically-quiescent in the normal state, it is one of the most rapidly repairing of all mammalian tissues (reviewed by [21]). The mechanism(s) involved in regulating the balance between urothelial differentiation and regeneration represent a paradox with major implications for urological practice.

There is a clinical need to reconstruct the lower urinary tract in patients with end-stage bladder diseases, including cancer (reviewed by [14]). In current surgical practices where the bladder is reconstructed with bowel (enterocystoplasty or cystectomy with urinary diversion using bowel), the large majority of complications arise from the incompatibility of bowel epithelium to long-term urine exposure [13], [15], [17]. Alternative tissue engineering strategies for bladder replacement and lower urinary tract reconstruction are at different stages of development and translation [2], [22], [40]. In all approaches, the need for a functional urinary barrier is paramount to avoid serious clinical complications. As a patient's own urothelium may be compromised by disease or the disease environment [35], the ability to generate urothelium from induced pluripotent or other autologous cells is an essential, albeit future goal.

We have previously described a cell culture system for propagating normal human urothelial (NHU) cells in low calcium, serum-free medium [33]. In these conditions, urothelial cells lose expression of archetypal transitional epithelial markers (including cytokeratin (CK) 13, differentiation-restricted CK20 and most uroplakin genes) and adopt a more primitive or “basal-like” EGFR-autocrine-regulated CK14-positive squamous epithelial cell phenotype [41]. Such cells retain capacity for urothelial-type differentiation, inducible either by ligand-activation of the nuclear receptor peroxisome proliferator-activated receptor gamma (PPARγ) [42], [43], [45], or by growth in serum [8]. Applied to bladder or ureteric human urothelial cells, both methods promote a common network of transcription factors (TFs) [4] that further induce the differentiation-associated expression of urothelial cytokeratins, tight junction claudins and uroplakins [42], [43], [44]. Specified TFs of the network, including interferon regulatory factor 1 (IRF1), forkhead box protein A1 (FOXA1), E74-like factor 3 (ELF3) and GATA Binding Protein 3 (GATA3), have been verified to have a functional role in transactivating the downstream differentiation-associated genes [12], [4], [45]. Further evidence of a causal influence on urothelial programming is the finding that many of these same TF genes are implicated in urothelial cancer [11], [38], [6].

It is axiomatic that the precise nature of the transcription factor networks will specify the differentiated phenotype. Therefore, we sought to identify key urothelium-determining TFs by comparing to oral epithelial cells from buccal mucosa. We proposed to use the identified constitutive differences in TF expression to perform a preliminary investigation into the influence of ectopic TF expression on the interconversion of oral epithelial into urothelial cells. As in other systems, where a transdifferentiation approach has informed the master cell type regulators (eg myogenic differentiation (MyoD) [9]), such an approach is predicted to identify the key factors required for urothelial conversion from pluripotent stem cell sources.

## 2 Materials and methods

### 2.1 Tissue samples

The collection and use of human tissues for research was covered by NHS Research Ethics Committee approvals, with patient informed consent as required. Buccal mucosa tissue samples were obtained as trimmings from male patients (age 3–65) undergoing urethral repair surgery. Urothelial samples (from renal pelvis or ureter) were obtained from anonymous male and female donors (age 1–79 years) undergoing a variety of urological procedures, including uninvolved urothelial tissue distal to renal cell carcinoma. Histological analysis on representative fragments was performed to check that all tissue samples were of normal morphology.

### 2.2 Cell culture

Normal human buccal epithelial (NHB) cells and normal human urothelial (NHU) cells were isolated as described [27], [33] respectively. In brief, to isolate NHB cells, first the epithelium was separated from the underlying connective tissue by incubation in 0.5% (w/v) dispase II (Roche) at 37 °C for 3–4 h. The epithelium was dissected into small pieces with scissors and the cells dissociated by incubation in a trypsin-EDTA solution at 37 °C for 5 min. For NHU cell isolation, tissue samples were incubated in an EDTA containing solution for 4 h at 37 °C. The epithelium, following separation from the underlying connective tissue using forceps, was incubated in 100 U/ml collagenase (type IV) for 20 min at 37 °C. Following the isolations, primary NHB and NHU cells were cultured identically on Primaria™ plasticware (Corning) in keratinocyte serum-free medium (KSFM) containing bovine pituitary extract (BPE) and epidermal growth factor (EGF) (Invitrogen) plus 30 ng/ml cholera toxin. NHB and NHU cell cultures were passaged at just-confluence and maintained as serially-passaged finite cell lines, as previously described for NHU cells [33]. Each experiment was performed on one to four independent donor cell lines, with a total of 16 independent NHB donor cell lines and 10 independent NHU donor cell lines used to perform the experiments described herein. The number (n) of independent cell lines used for each experiment is provided in the respective figure legends.

### 2.3 Urothelial differentiation protocols

Two protocols optimised previously for the differentiation of NHU cells were applied for the differentiation experiments reported here; NHU cells cultured identically to the NHB cells were included as comparative controls.

First, to induce differentiation by PPARγ activation, 70% confluent epithelial (NHU control or NHB) cell cultures were exposed to 1 µM PD153035 (EGFR-TK inhibitor) (Merck Millipore) and 1 µM troglitazone (TZ; PPARγ agonist) (Tocris) for 24 h, before replacement with 1 µM PD153035 alone (protocol termed TZ/PD) [43].

Second, to produce stratified epithelial cell sheets, epithelial (NHU control or NHB) cell cultures were grown to 80% confluence and the medium was changed to contain 5% (v/v) adult bovine serum (ABS; SeraLab) for five days. For Trans-Epithelial Electrical Resistance (TEER) studies, the cultures were then passaged and seeded onto 12 mm, 0.4 µm pore-size Snapwell culture inserts (Corning^®^ Costar^®^). The insert cultures were established in medium containing 5% ABS for 24 h and the medium was changed to contain 5% ABS plus 2 mM [Ca^2+^] [8].

### 2.4 Barrier assessment and cell sheet harvest

TEER measurements were taken using chopstick STX2 electrodes and an Epithelial Voltohmeter (World Precision Instruments). The average TEER of Snapwell culture inserts containing medium only was subtracted from each recorded value. To harvest epithelial cell sheets from the Snapwell culture inserts, cell sheets were incubated with 2% (w/v) Dispase II (Roche) for 30 min at 37 °C. Released cell sheets were floated into CellSafe+ biopsy capsules (CellPath) and placed into embedding cassettes. The cell sheets were fixed in 4% formaldehyde for 24 h, before processing into paraffin wax.

### 2.5 Antibodies

The following primary antibodies were used at the stated titrations for immunoblotting (IB), immunofluorescence (IF) and immunohistochemistry (IHC): β-actin (ACTB; Sigma-Aldrich, AC-15) (IB – 1:250,000); claudin 3 (Life Technologies, Z23.JM) (IB – 1:4000); claudin 4 (Life Technologies, 3E2C1) (IB – 1:1000); claudin 5 (Life Technologies, 4C3C2) (IB – 1:1000); claudin 7 (Life Technologies, ZMD.241) (IB – 1:1000); cytokeratin 5 (CK5) (Abcam, SP27) (IHC – 1:100); cytokeratin 5 (CK5) (The Binding Site, PH607) (IF – 1:100); cytokeratin 7 (CK7) (Novocastra, OV-TL12/30) (IHC – 1:400, IF – 1:40); cytokeratin 13 (CK13) (Abnova, 1C7) (IHC – 1:500, IF – 1:500); cytokeratin 14 (CK14) (Serotec, LL002) (IHC – 1:1200); cytokeratin 14 (CK14) (ICRF, LL001) (IF – 1:5); cytokeratin 20 (CK20) (Novocastra, Kr20.8) (IHC – 1:200); Cytokeratin 20 (CK20) (Cymbus Bioscience, IT-Ks20.3) (IF – 1:100); ELF3 (Abcam, EPESER1) (IF – 1:1000, IB – 1:20,000); FOXA1 (Santa Cruz Biotechnology, Q-6) (IF – 1:200, IB – 1:500); FOXA1/2 (Santa Cruz Biotechnology, C-20) (IF – 1:200); GATA3 (Cell Signaling, D13C9) (IF – 1:800, IB – 1:1000); PPARγ (Cell Signaling, D69) (IF – 1:100, IB – 1:500).

### 2.6 Immunohistochemistry (IHC)

5 µm sections were dewaxed through xylene into ethanol. Antigen retrieval was performed by boiling the slides in 10 mM citric acid buffer (pH 6.0) for 10 min. For some antibodies (CK13 and CK14), cell sheets were incubated in 0.1% trypsin at 37 °C prior to the citric acid incubation. Sections were blocked using an avidin/biotin blocking kit (Vector Laboratories) and 5% rabbit or goat serum (as determined by the secondary antibody), incubated with primary antibody at 4 °C overnight, then washed. A biotinylated secondary antibody was applied to the cell sheets, followed by a streptavidin-biotinylated/horseradish peroxidase complex (Vector Laboratories), before visualisation with diaminobenzidine (Sigma Aldrich). The cell sheets were counterstained lightly with haematoxylin, dehydrated and mounted in DPX (CellPath). Appropriate methodology, specificity and negative controls were included for each antibody.

### 2.7 Reverse Transcribed-Polymerase Chain Reaction (RT-PCR) and quantitative RT-PCR (RT-qPCR)

Cell cultures were solubilised using TRIzol™ Reagent and RNA extraction was performed using a phenol-chloroform and isopropanol precipitation method according to the manufacturer's instructions (Invitrogen). Any contaminating genomic DNA was removed by DNase digestion (DNA-free™, Ambion) and checked using RT-negative controls. cDNA was synthesized from 1 µg of total RNA using the first-strand synthesis system primed with random hexamers (Invitrogen). RT-PCR and RT-qPCR were performed as described previously [4]. A list of PCR primers is provided in Table 1. RT-qPCR was performed using three technical replicates for each gene. RT-PCR was performed using 30 cycles except for *GAPDH* (25 cycles).

**Table 1:** List of primers used for RT-PCR and RT-qPCR.

**Gene**	**Forward primer (5′-3′)**	**Reverse primer (5′-3′)**	**Use**
*ELF3*	GTTCATCCGGGACATCCTC	GCTCAGCTTCTCGTAGGTC	RT-PCR
*ELF3*	TCAACGAGGGCCTCATGAA	TCGGAGCGCAGGAACTTG	RTqPCR
*FOXA1*	CAAGAGTTGCTTGACCGAAAGTT	TGTTCCCAGGGCCATCTGT	RT-PCR
			RTqPCR
*GATA3*	TCCAGACACATGTCCTCCCT	TGGTGTGGTCCAAAGGACAG	RT-PCR
*GATA3*	TCTATCACAAAATGAACGGACAGAA	TGTGGTTGTGGTGGTCTGACA	RTqPCR
*GRHL3*	GTGACAAGGGAGCTGAGAGG	CAGTCTCTGGCCGAAGGTAG	RT-PCR
*IRF1*	GCTGGGACATCAACAAGGAT	GTGGAAGCATCCGGTACACT	RT-PCR
*KLF5*	GACACCTCAGCTTCCTCCAG	ACTCTGGTGGCTGAAAATGG	RT-PCR
*PPARG*	AGACAACCTGCTACAAGCCC	GGAAATGTTGGCAGTGGCTC	RT-PCR
*PPARG*	GAACAGATCCAGTGGTTGCAG	CAGGCTCCACTTTGATTGCAC	RTqPCR
*UPK1A*	GGGGTATCTCGTGGTTTGGG	CGTAAGGGCTAGGGACGTTG	RT-PCR
*UPK1A*	CATTCTTGCTGAACCGTTTGTG	GTGACCGTGACAGAACTCTCATG	RTqPCR
*UPK1B*	TTGAAGCCACCGACAACGAT	AACAGACAGGCAGAAGAGGC	RT-PCR
*UPK1B*	CGCTTGCCTTCAGCTTGTG	GGCCCTGGAAGCAACGA	RTqPCR
*UPK2*	CTCCCGCAAGTAAGGAGGT	GAAGGATGGGGGAATTGTTA	RT-PCR
*UPK2*	CAGTGCCTCACCTTCCAACA	TGGTAAAATGGGAGGAAAGTCAA	RTqPCR
*UPK3A*	ATGGGGAGTTCTGATGGGGA	TGCTGGAATACACCTCAGCC	RT-PCR
*UPK3A*	CGGAGGCATGATCGTCATC	CAGCAAAACCCACAAGTAGAAAGA	RTqPCR
*UPK3B*	CCTCCTGCTTCACTCTCTCTGTCT	GAAACTGACAATCACGGCAGAA	RT-PCR
			RTqPCR
*GAPDH*	CAAGGTCATCCATGACAACTTTG	GGGCCATCCACAGTCTTCTG	RT-PCR
			RTqPCR

### 2.8 Immunoblotting

Whole protein lysates were generated from cell cultures using a reducing 2× SDS lysis buffer containing 1% protease inhibitor (Sigma-Aldrich). Lysates were incubated on ice for 30 min and centrifuged at 20,000*g* for 30 min at 4 °C. 25 µg was loaded into either 4–12% bis-Tris gels or 3–8% Tris-acetate gels (Novex®) and electrophoresis was performed at 200 V for 1 h. Proteins were transferred to an Immobilon-FL 0.45 µm PVDF membrane by electroblotting. Membranes were blocked using Odyssey Blocking Buffer (Li-Cor) and incubated with the primary antibody overnight at 4 °C. The fluorescent secondary antibody was applied to the membrane for 1 h at ambient temperature, and membranes were imaged for semi-quantification using an Odyssey® infrared imaging system (Li-Cor).

### 2.9 Immunofluorescence microscopy

Cells were cultured on 12-well glass slides (C A Hendley Essex Ltd), fixed in 4% formaldehyde for 10 min and permeabilised with 0.1% Triton™ X-100 (Sigma Aldrich), before incubation with primary antibody in a 0.1% BSA solution overnight at 4 °C. A fluorescent-conjugated secondary antibody was applied to the cells for 1 h at ambient temperature, before further washing and counterstaining of nuclei with 0.1 µg/ml Hoechst 33258 (Sigma Aldrich).

### 2.10 Overexpression of GATA3 and PPARγ1 in NHB cells by retroviral transduction

GATA3 and PPARG overexpression was achieved by cloning consensus coding sequences for full-length GATA3 protein (CCDS31143) and the PPARγ1 protein variant (termed "PPARG1" throughout; CCDS2610) into the retroviral vector pLXSN (Clontech) and verified by Sanger sequencing. The pLXSN-GATA3 and pLXSN-PPARG1 plasmids were transfected into PT67 retrovirus packaging cells (Clontech) and selected using G418. NHB cells were transduced with conditioned medium from PT67 cells containing replication-defective retrovirus and selected using G418. Control NHB cells were transduced with the pLXSN vector only (Empty).

### 2.11 Statistical analysis

Statistical analysis was performed where appropriate using either a two-tailed, paired *t*-test or a one-way ANOVA with post-test. Error bars represent standard deviation.

## 3 Results

### 3.1 Comparison of human urothelial and buccal epithelial phenotypes *in situ* and *in vitro*

**Fig. 1:**
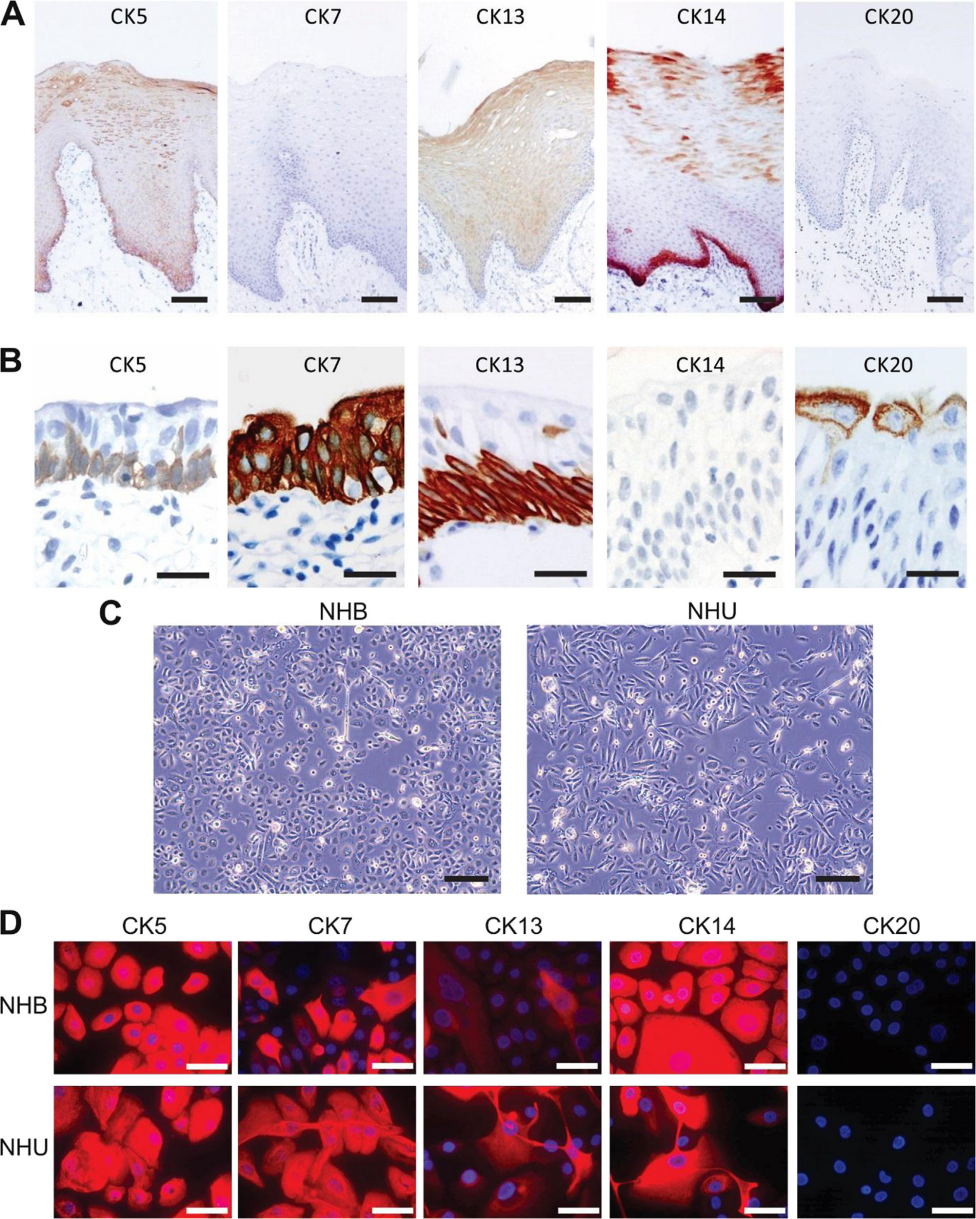
**Comparison between buccal epithelium and urothelium**. (A-B) Histological sections showing representative *in situ* immunolocalisation patterns for cytokeratins CK5, CK7, CK13, CK14 and CK20 in (A) buccal mucosa (scale bar ≡ 100 µm) and (B) urothelium (scale bar ≡ 25 µm). (C) Representative phase contrast images of NHB and NHU cells grown *in vitro* (scale bar ≡ 200 µm). (D) Immunofluorescence microscopy images of cytokeratin CK5, CK7, CK13, CK14, and CK20 expression by NHB and NHU cells grown in low calcium, serum-free medium (KSFMc). Immunolabelling was performed on n = 3 independent NHB cell lines and images are representative, although note that CK13+ cells are infrequent in NHU cell cultures grown in these non-differentiated conditions. Scale bar ≡ 50 µm.

*In situ*, the epithelium of buccal mucosa is a non-cornified stratified squamous epithelium that can average 30 cell layers thick. By contrast, the urothelium, a transitional epithelium, averages 4–5 cell layers. Immunohistochemical examination of CK5, CK7, CK13, CK14 and CK20 revealed expression of CK5 in all layers of buccal epithelium and an absence of CK7 and CK20 expression. CK13 expression was suprabasal, whilst CK14 was particularly intense but not exclusive to the basal layers of buccal epithelium (Fig. 1A). In urothelium, CK5 and CK13 were associated with the basal layers, CK7 was present in all layers, CK14 was absent and CK20 was confined to the superficial cell layer (Fig. 1B).

When isolated and maintained in identical low calcium [0.09 mM] serum-free culture conditions (Fig. 1C), both NHU and NHB cells formed proliferative, contact-inhibited monolayer cultures that upon reaching confluence could be serially sub-cultured up to 10 times (data not shown). The *in vitro* expression of cytokeratin proteins by both cell types was similar by immunocytochemistry, with CK5, CK7, CK13 and CK14 detected, including gain of CK7 by NHB cells and gain of CK14 by NHU cells; CK20 was not expressed (Fig. 1D).

### 3.2 Generation of cell sheets and measurement of barrier function

**Fig. 2:**
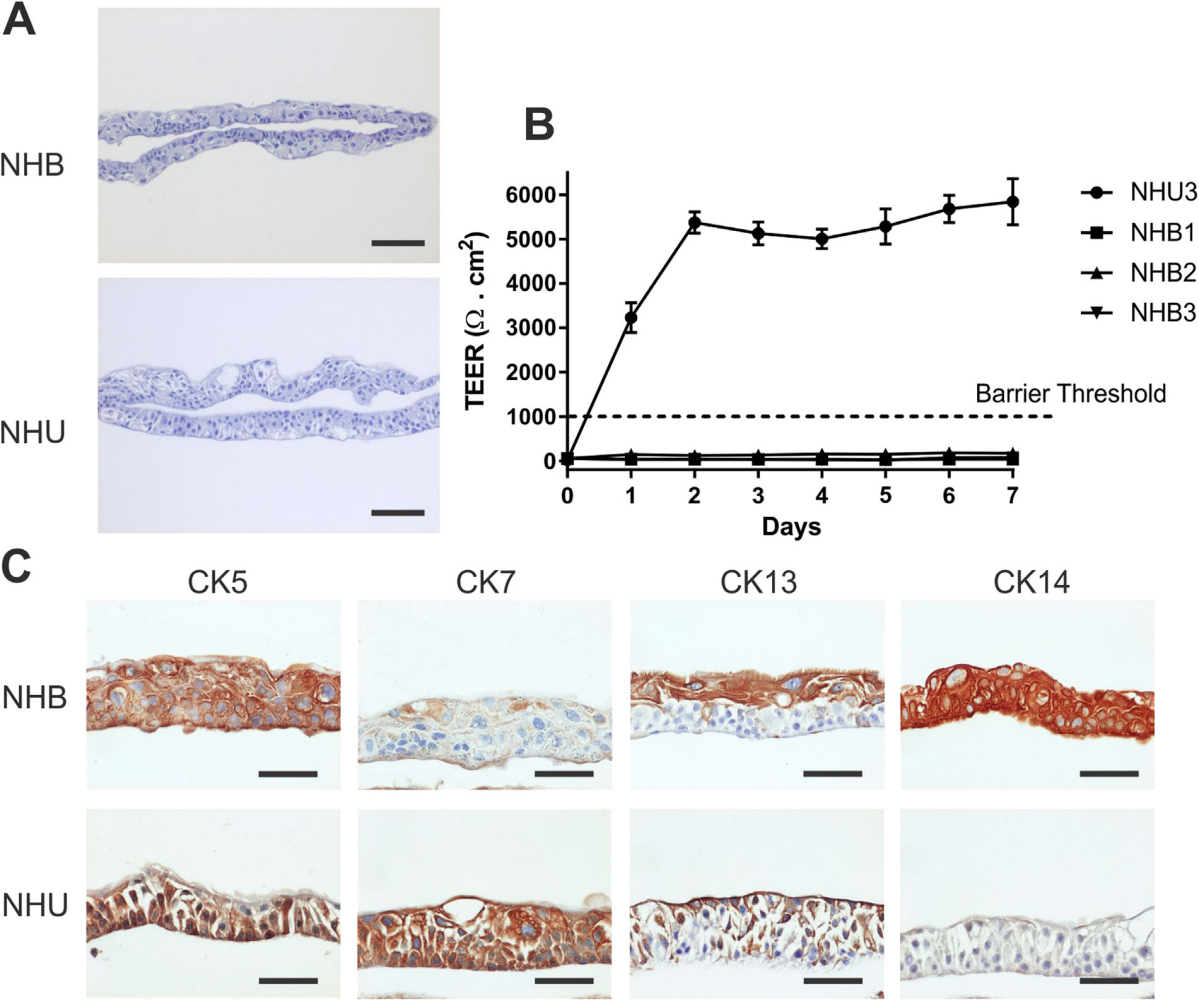
**Formation of cell sheets and barrier function.** The ability to form a stratified barrier epithelium was examined in three independent NHB cell lines, with a representative NHU cell line provided for comparison purposes. (A) Representative haematoxylin and eosin-stained NHB and NHU cell sheets showing multi-layered tissue structures formed 7 days post-seeding onto membranes in serum- and 2 mM calcium-containing medium. Scale bar ≡ 100 µm. (B) Trans-epithelial electrical resistance (TEER) measurements taken daily. Day 0 measurements were taken 24 h after seeding the cells onto membrane inserts, directly before the medium was changed to increase the calcium concentration to 2 mM. (C) Immunohistochemical analysis of NHB and NHU cell sheets for CK5, CK7, CK13 and CK14 protein expression. Representative results shown from experiments performed on n = 3 independent NHB cell lines. Scale bar ≡ 50 µm.

Using a protocol optimised for differentiated barrier induction by NHU cells in vitro [8], NHB cultures formed multi-layered cell sheets that were similar morphologically to those achieved by NHU cells cultured in identical conditions (Fig. 2A). Using TEER to assess barrier function, NHB cell sheets were unable to form a tight barrier (defined here as ≥ 1 kΩ .cm^2^), compared to typical barriers formed by NHU cells of 3–5 kΩ. cm^2^ (Fig. 2B). Immunohistochemical analysis of cytokeratin expression in NHB cell sheets demonstrated consistent expression of CK5 and CK14 throughout all layers, with CK13 limited to the upper portion of the cell sheets, and diffuse, weak CK7 expression (Fig. 2C). By contrast, NHU cell sheets were CK7-positive throughout all cell layers and demonstrated reciprocal patterns of CK5 and CK13, but were negative for CK14.

### 3.3 Expression of Uroplakin (*UPK*) Genes by NHB cells *in vitro*

**Fig. 3:**
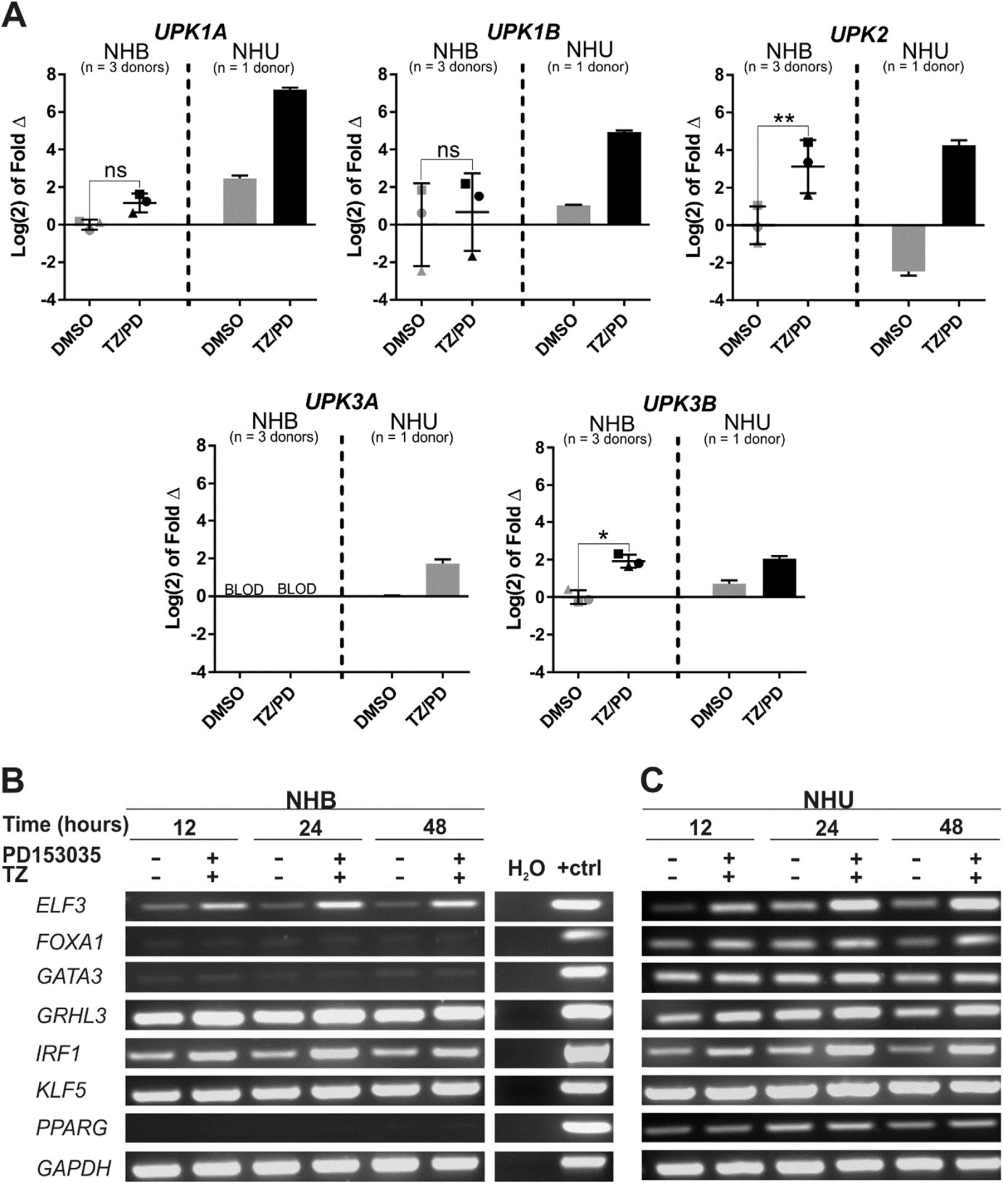
**Comparison of uroplakin and urothelium differentiation-associated transcription factor gene expression by NHB and NHU cell cultures.** Employing protocols developed to differentiate NHU cells by PPARγ activation, cell cultures of NHB or NHU cells were exposed to 1 µM troglitazone and 1 µM PD153035 (TZ/PD) for 24 h, maintained in 1 µM PD153035 and harvested at 12, 24, 48 and/or 72 h. Control cultures were exposed to vehicle (0.1% DMSO) alone. (A) RTqPCR for three independent NHB cell lines (represented by different symbols), versus a single NHU cell line for comparison of uroplakin (UPK1A, UPK1B, UPK2, UPK3A and UPK3B) mRNA expression at the 72 h time-point. All data has been normalised to *GAPDH* expression and is presented relative to the DMSO-treated NHB cells for each gene except *UPK3A,* where the data is shown relative to the DMSO treated NHU cells due to absent *UPK3A* gene expression by NHB cells. BLOD = Below Limit of Detection. Statistical analysis was performed using a two-tailed, paired *t*-test to determine whether TZ/PD resulted in any significant change in gene expression in NHB cells. *represents P ≤ 0.05, ** represents P ≤ 0.01. Error bars represent standard deviation. (B-C) RT-PCR of *ELF3, FOXA1, GATA3, GRHL3, IRF1, KLF5 and PPARG* mRNA expression by (B) NHB cells and (C) NHU cells. RNA was extracted at the 12, 24 and 48 h time-points and then DNAase-treated and used to generate cDNA for RT-PCR. *GAPDH was* used as an internal loading control. A no-template (H_2_O) control was included as a negative control for the PCR reaction, and genomic DNA was used as the positive control (+ctrl). No product was amplified from RT-negative controls (not shown). Experiments were performed on n = 2 independent NHB donor cell lines and representative results shown.

Uroplakin transcript expression was evaluated initially by RT-PCR in control NHB cell cultures and following combined TZ/PD treatment (Fig. S1) [43]. NHB cells showed robust expression of *UPK1B* and *UPK3B,* with equivocal expression of *UPK1A* and *UPK2* appearing at later (confluence-associated) time-points in both baseline and induced conditions (Fig. S1). These results were confirmed by RT-qPCR in three independent NHB donor cell lines, where NHB cells were shown to express transcripts for *UPK1A*, *UPK1B*, *UPK2* and *UPK3B* (Fig. 3A). The expression of *UPK2* and *UPK3B* transcripts by NHB cells was significantly upregulated at 72 h following combination TZ/PD therapy (Fig. 3A). Expression of *UPK3A* was absent in NHB cells.

### 3.4 Expression of urothelial differentiation-associated transcription factors by NHB cells in vitro

Based on the clear phenotypic differences observed between the two cell types *in vitro*, RT-PCR was used to evaluate expression of seven transcription factor genes (*ELF3, FOXA1, GATA3, GRHL3, KLF5, IRF1* and *PPARG*) implicated in urothelial development and/or differentiation [3], [4], [42], [43], [44], [45], [47] (Fig. 3B&C). NHB cells showed absent or barely-detectable expression of *FOXA1*, *GATA3* and *PPARG* transcripts even following attempts using TZ/PD to initiate differentiation in case of a positive feedback loop. Expression of *GRHL3*, *IRF1* and *KLF5* appeared constitutive in nature, while *ELF3* expression was upregulated in response to TZ/PD.

**Fig. 4:**
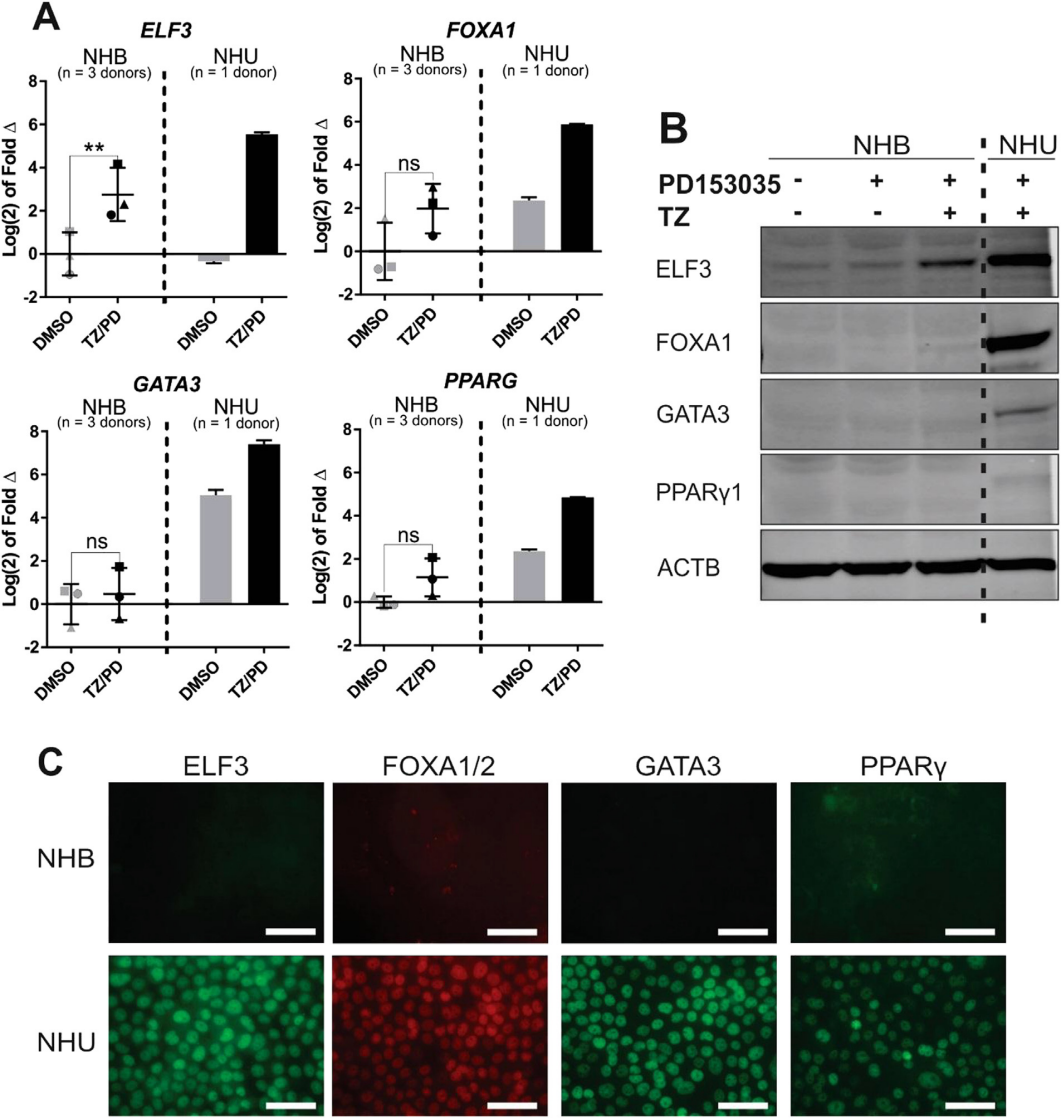
**Evaluation of *ELF3*, *FOXA1*, *GATA3* and *PPARG* expression in NHB cells.** RNA and protein were extracted from parallel cultures of NHB and NHU cells at 72 h following exposure to the PPARγ-activating TZ/PD protocol, or a vehicle control (0.1% DMSO). (A) RTqPCR results combined from three independent NHB cell lines (represented by different symbols), with a single NHU cell line for comparison. All data is normalised to *GAPDH* expression and is presented relative to the DMSO-treated NHB cell control for each gene. Statistical analysis was performed using a two-tailed, paired *t*-test to test if TZ/PD treatment resulted in any significant change in gene expression in NHB cells. ** represents P ≤ 0.01. Error bars represent standard deviation. (B) Immunoblot of whole cell protein lysates from representative NHB and NHU cell cultures following exposure to the TZ/PD protocol, 1 µM PD153035 alone, or vehicle (0.1% DMSO) for 72 h. ACTB was included as an internal loading control. Experiments performed on n = 3 independent NHB cell lines with similar results. (C) Immunofluorescence microscopy of ELF3, FOXA1/2, GATA3 and PPARγ in representative NHB and NHU cell cultures. Images taken at identical exposures to demonstrate differences in labelling intensity between the two cell types. Experiments performed on n = 3 independent NHB donor cell lines with similar results. Scale bar ≡ 50 µm.

RTqPCR analysis of three independent NHB donor cell lines confirmed the results obtained by RT-PCR, demonstrating weak expression of *FOXA1*, *GATA3* and *PPARG*, with significant upregulation only of *ELF3* transcript following TZ/PD treatment (Fig. 4A). Similar results were obtained by evaluating protein expression by western blotting (Fig. 4B). By immunofluorescence microscopy, ELF3, FOXA1/2, GATA3 and PPARγ were weak/absent in NHB cells treated with combined TZ/PD, contrasting to the typically strong nuclear protein expression seen when NHU cells were subjected to identical conditions *in vitro* (Fig. 4C).

### 3.5 NHB overexpression studies

To further investigate transcription factor regulation and hierarchies, full-length protein coding sequence for GATA3 and PPARγ1 were selected to stably overexpress in NHB cells due to their low/absent expression in NHB cells, and known expression and importance in urothelial differentiation. PPARγ1 was selected over PPARγ2 due to previous evidence suggesting that PPARγ1 is the primary *PPARG* isoform required for urothelial cell-type differentiation [34].

### 3.6 GATA3

**Fig. 5:**
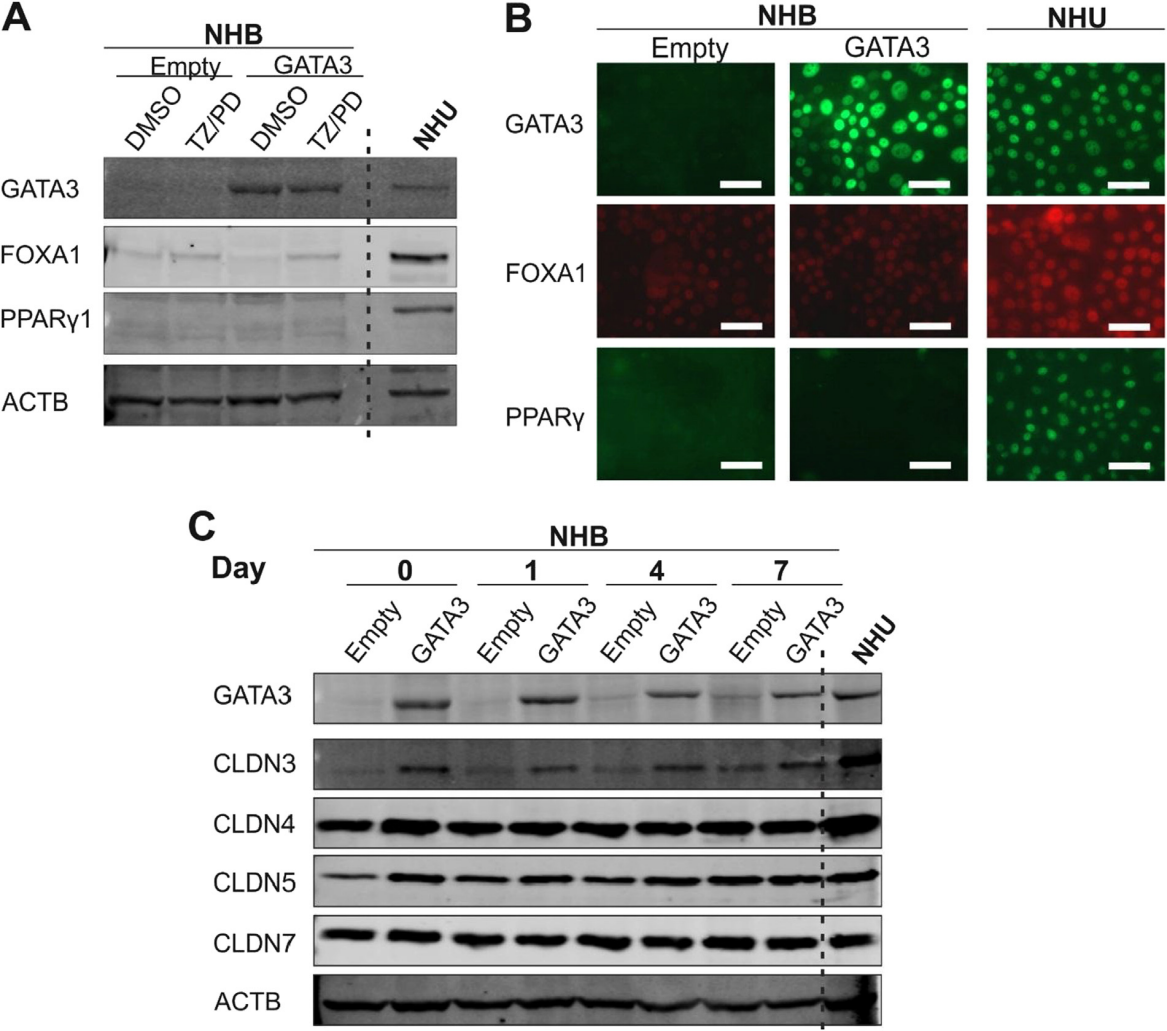
***GATA3* overexpression in NHB cells.** (A) *GATA3* overexpressing and control (empty vector) NHB cell cultures following exposure to the PPARγ-activating TZ/PD protocol for 72 h. Western blotting of whole protein lysates was performed to assess protein expression of GATA3, FOXA1 and PPARy1. NHU cells (non-transduced) and treated with the TZ/PD protocol for 72 h are shown for comparison. (B) *GATA3* overexpressing and control (empty vector) NHB cells at 72 h post TZ/PD protocol. GATA3, FOXA1 and PPARy protein expression assessed by indirect immunofluorescence microscopy. NHU cells (non-transduced; 72 h TZ/PD protocol) were included as positive controls for comparison. Scale bar ≡ 50 µm. (C) *GATA3* overexpressing and control (empty vector) NHB cells were induced to form cell sheets using 5% ABS and 2 mM calcium for up to 7 days. Expression of the tight junction-associated proteins, claudin 3, 4, 5 and 7, assessed by western blotting. ACTB was included as a loading control. NHU cells (non-transduced) exposed to the same protocol were used as a positive control for comparison. Experiments were performed on n = 2 independent NHB donor cell lines and representative results shown.

Successful overexpression of GATA3 was achieved in NHB cells and resulting in nuclear immunolocalisation (Fig. 5A&B). Overexpression of GATA3 did not cause any noticeable effect on FOXA1 or PPARγ1 protein expression in NHB cells, as shown by both western blotting and immunofluorescence microscopy (Fig. 5A&B). This remained true even following attempts to activate PPARγ by combined TZ/PD treatment. Expression of tight junction-associated proteins was examined when transduced NHB cells were induced to form cell sheets using 5% bovine serum and 2 mM [Ca^2+^]. A clear upregulation of claudin 3 protein expression was observed following GATA3 overexpression in NHB cells (Fig. 5C). Expression of other urothelium-associated tight junction proteins, claudins 4, 5 and 7 was observed consistently in both control (Empty vector) and GATA3 overexpressing NHB cells. GATA3 overexpression, and subsequent claudin 3 upregulation, failed to result in the gain of barrier function by NHB cells sheets, as measured by TEER (81.74 ± 2.35 Ω cm^2^, Day 4, n = 3; Control: 110.36 ± 3.54 Ω cm^2^, Day 4, n = 3).

### 3.7 PPARG1

**Fig. 6:**
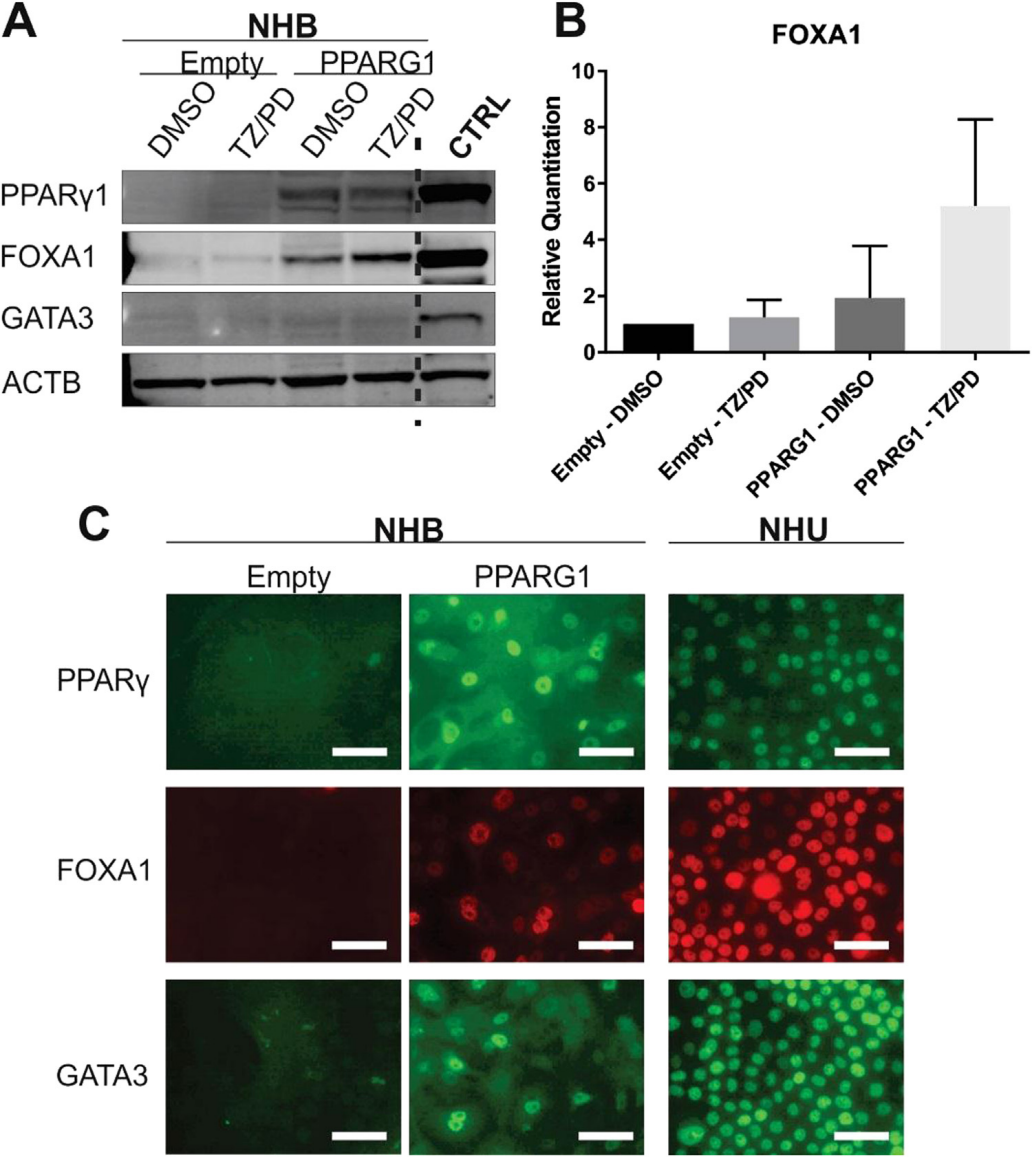
**Evaluation of PPARγ, FOXA1 and GATA3 expression in PPARG1 overexpressing and control (empty) NHB cells.** PPARG1 overexpressing and control (empty vector) NHB cell cultures were exposed to the TZ/PD protocol for 72 h. Experiments performed on between 2 and 4 independent NHB cell lines (as stated below), with representative results shown. (A) PPARγ1, FOXA1 and GATA3 protein expression assessed by western blotting. ACTB expression was included as an internal loading control. Protein lysates from cell lines known to express the proteins of interest were included as positive controls for each antibody (CTRL). Experiments performed on n = 3 independent NHB cell lines. (B) Densitometry analysis of FOXA1 protein expression shown relative to control (Empty - DMSO) NHB cells. Data is shown as the mean of n = 4 independent transduced NHB cell lines. All values were normalised to the ACTB expression. Statistical analysis was performed using a one-way ANOVA test, but no statistical significance was found (P > 0.05). Error bars represent standard deviation. (C) Immunofluorescence microscopy evaluating PPARγ, FOXA1 and GATA3 protein localisation in PPARG1 overexpressing and control (empty vector) NHB cells following the TZ/PD protocol at 72 h. Experiments were performed on n = 3 independent transduced NHB cell lines. IF images for a single NHB cell line are shown. Nuclear localisation was observed with PPARγ (n = 3/3), FOXA1 (n = 3/3), and GATA3 (n = 2/3). NHU cells (non-transduced) treated with the TZ/PD protocol are shown for comparison at the same time point. Scale bar ≡ 50 µm.

Overexpression of PPARγ1 protein coding sequence (PPARG1) in NHB cells caused a noticeable increase in FOXA1 protein expression by western blotting (Fig. 6A), although this was not significant when quantified across transductions in four independent NHB lines (Fig. 6B). GATA3 protein expression was poorly detectable by western blotting in PPARG1 overexpressing NHB cells, even following attempts to activate PPARγ using combination TZ/PD treatment. However, by immunofluorescence microscopy PPARG1 overexpression resulted in clear *de novo* nuclear localisation of PPARγ (n = 3/3), FOXA1 (n = 3/3) and GATA3 (n = 2/3) (Fig. 6C).

**Fig. 7:**
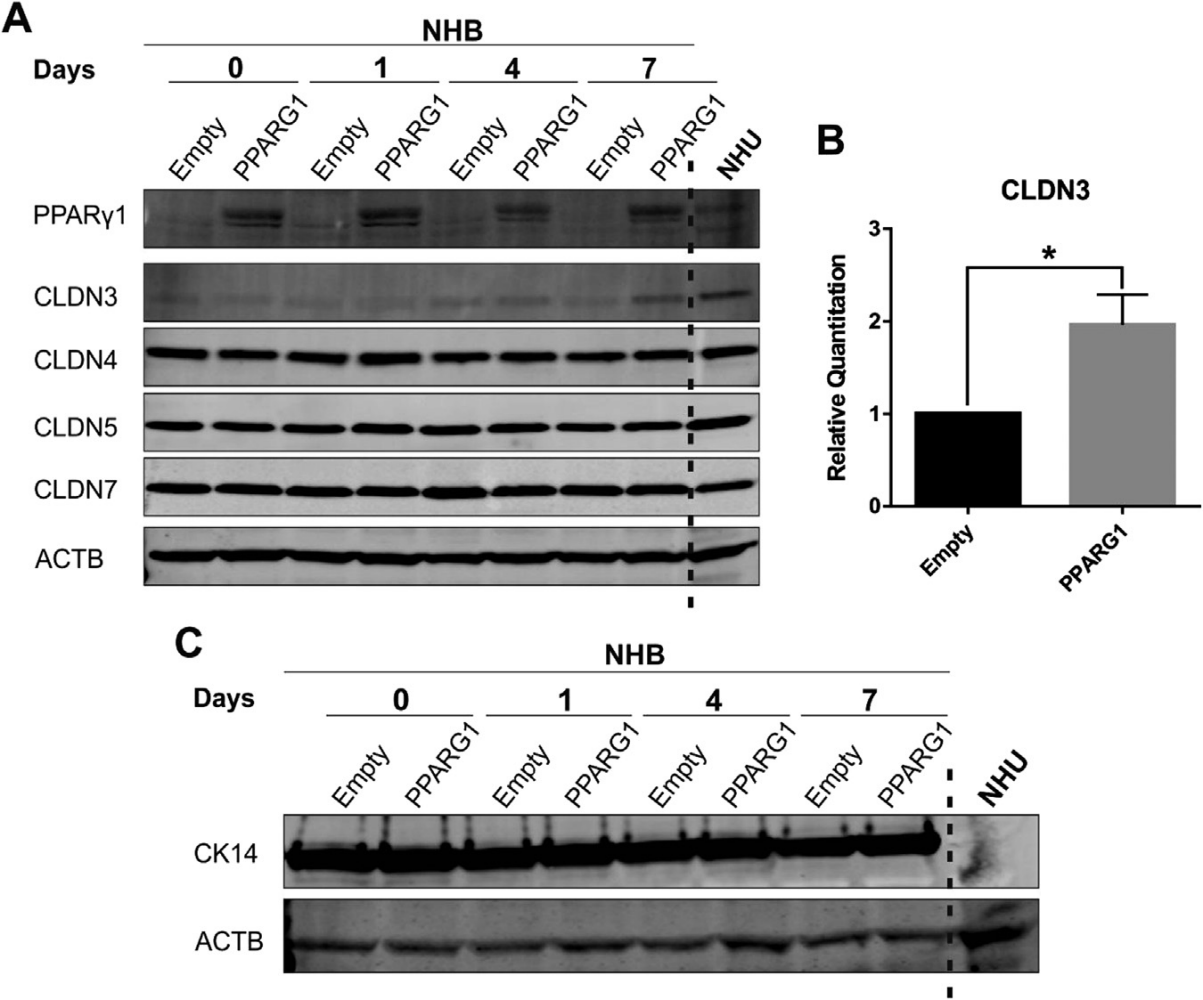
**Assessment of tight junction-associated proteins and CK14 expression in PPARG1 overexpressing and control (empty) NHB cells.** PPARG1 overexpressing and control (empty vector) transduced NHB cells were induced to stratify using serum and calcium (detailed in Section 2). Experiments were performed on n = 3 independent NHB donor cell lines. Results at 7 days shown for a representative transduced NHB cell line. ACTB expression was used as internal loading control. NHU cells (non-transduced) exposed to the same protocol for 5 days are shown for comparison. (A) Evaluation of tight junction-associated protein expression by western blotting with claudins 3, 4, 5 and 7. (B) Densitometry analysis of claudin 3 expression at day 7 shown relative to control (empty vector) cells. All values were normalised to the ACTB expression. Data is shown as the mean of n = 3 independent transduced NHB donor cell lines. Statistical analysis performed using a two-tailed, paired *t*-test. * represents P ≤ 0.05. Error bars represent standard deviation. (C) CK14 expression evaluated by western blotting.

Evaluation of tight junction-associated proteins following stratification of transduced NHB cells, revealed a significant upregulation of claudin 3 protein expression (Fig. 7A&B). The expression of claudins 4, 5 and 7 remained consistently expressed in both control (empty vector) and PPARG1-overexpressing cells (Fig. 7A). In addition, PPARG1 overexpression had no effect on CK14 expression, which was detectable in both control (empty vector) and PPARG1 overexpressing NHB cells (Fig. 7C). Finally, PPARG1 overexpression did not result in the gain of barrier function in NHB cell sheets, as measured by TEER (54.24 ± 2.99 Ω cm^2^, Day 4, n = 3; Control: 15.82 Ω cm^2^, Day 4, n = 2).

## 4 Discussion

In this study we have used a different epithelial cell type in experiments aimed at informing future strategies for programming autologous cells to generate human urothelium. Our study has shown that PPARγ1 and GATA3 are nuclear factors differentially expressed by human urothelial, not buccal, epithelial cells and that when expressed as individual transgenes, these factors effect specific changes on buccal epithelial cell phenotype.

Various groups have attempted to generate human urothelial cells from different types of stem cells in order to identify a surrogate cell source for autologous bladder tissue engineering (reviewed by [5]). The initiating human cell sources investigated have included bone marrow-derived mesenchymal stem cells [39]; adipose-derived stem cells [31]; amniotic fluid-derived stem cells [19]; umbilical cord stem cells [46]; pluripotent embryonic stem cells [29]; induced pluripotent stem cells (iPSCs) [20], [24]; and voided urine cells [48]. The inducing method has frequently assumed production of unidentified instructive paracrine factors by urothelial or bladder cancer cell lines employed in co-culture [23], [26], [39], [7], or via medium-conditioning [19], [24], [31], [46], [49]. Collectively, the results suggest that from any naïve heterotypic starting population, an unknown proportion of cells will express urothelial differentiation-associated genes under permissive conditions.

More systematic attempts have used a two-staged approach in which pluripotent stem cells of embryonic [29] or iPSC [20] derivation are first differentiated into definitive endoderm cells. This is followed by attempts to direct urothelial differentiation using agonists that activate PPARγ [29] or the retinoic acid receptor (RAR) [20], as candidate nuclear receptors whose activation is implicated in urothelial differentiation.

Irrespective of the approach, all above studies have reported qualitatively similar outcomes in detecting upregulated epithelial- and urothelial-associated gene expression within the emergent population. Fold increases in the expression of uroplakin (UPK) genes has been presented as *prima facie* evidence of urothelial conversion, even though it is well established that uroplakin transcripts are expressed by other (non-urothelial) epithelial cell types [1], [18], [28], [30]. UPK1B and UPK3B are the least differentiation stage-restricted of the uroplakins in urothelium and here, buccal epithelial cells *in vitro* were shown clearly to express both *UPK1B* and *UPK3B* transcripts. Transcripts for *UPK1A* and *UPK2*, which are more differentiation stage-restricted in urothelium, were also, albeit variably, expressed by buccal epithelial cells, further establishing that expression of uroplakin gene transcripts alone cannot be used to definitively mark urothelium-specific conversion. Only *UPK3A* expression was absent from native buccal epithelial cells *in vitro*, potentially indicating that *UPK3A* expression could represent a more objective marker of successful (re)programming to differentiated urothelium. This study further indicated the absence of CK14 and expression of CLDN3 as part of the urothelial signature phenotype.

We have previously shown that maintenance of NHU cells *in vitro* in a low calcium, serum-free medium results in the loss of urothelial phenotype and reversion to a more primitive squamous state, accompanied by changes in chromatin organisation [12]. Under identical serum-free culture conditions, buccal epithelial cells similarly adopt a proliferative, non-stratified CK14^+^ squamous phenotype. The provision of serum, in conjunction with physiological calcium to promote development of intercellular junctions and polarity, led to re-establishment of the original differentiation programmes of urothelial and buccal cells, respectively, indicating that despite the apparent plasticity, cultured cells perhaps retained an epigenetic “memory”. The addition of serum provides a permissive environment for differentiation, revealing underpinning differences in epithelial lineage programming, with urothelial cells switching to a CK13^+^ transitional programme, whereas buccal epithelial cells maintain the CK14^+^ stratified squamous programme.

A comparison of TF expression between buccal versus urothelial cells revealed differential expression of FOXA1, GATA3 and PPARγ. In preadipocytes, ligand activation of PPARγ protein provokes transactivation of *PPARG* as a differentiation-inducing positive feedback event [25]. This led us to investigate whether ligand-induced activation of PPARγ in wild type buccal epithelial cells could alone entrain a urothelial-like differentiation response as part of a positive feedback response. However, in the event, it failed to initiate expression of PPARγ, FOXA1 or GATA3, further supporting the idea that inherent or programmed differences between cells (rather than perhaps the presence of inducing ligands) defines lineage/differentiation potential. Whereas forced overexpression of *GATA3* alone failed to have any clear impact on the expression of PPARγ or FOXA1 in buccal epithelial cells, forced expression of *PPARG1* did promote both FOXA1 and GATA3 expression, indicating an upstream position in the regulatory network.

The primary function of the urothelium is to act as a barrier to urine and even following propagation *in vitro*, urothelium-derived cells retain the capacity to differentiate to form a functional barrier epithelium. We have shown here that when maintained under identical serum-containing culture conditions, *in vitro*-propagated buccal epithelial cells can form a stratified epithelial structure, whilst failing to form a functional barrier. The claudins are the main functional barrier-determining constituent of the tight junction, and claudin 3, which has been identified previously as a critical tight junction protein required (but not sufficient) for urothelial barrier function [32], was weakly expressed in wild type buccal epithelial cells. Our approach has demonstrated that individual overexpression of GATA3 and PPARγ1 are both able to promote increased claudin 3 protein expression in buccal epithelial cells, providing circumstantial evidence implicating them in the urothelial differentiation and barrier formation programme.

To date, no study has completely defined the conditions or factors required to direct pluripotent cells into coherent urothelial cells capable of forming a functional urinary barrier. This suggests a need for better criteria to inform and monitor the process of successful urothelial cell programming. Here, we have brought some clarity to the order of complex endogenous TF relationships likely to be operating in urothelial cells, where there is both interplay between TFs at the level of cooperativity and competition for DNA binding, along with positive and negative feedback on TF transcription regulation [12].

In conclusion, our results support PPARγ1 as a key upstream regulator that could constitute a component of a minimal transcription factor network required to promote urothelial-type differentiation. As buccal epithelial cells were shown to retain constitutive differences of phenotype in serum-free (non-permissive) culture conditions, we suggest that *PPARG1* overexpression experiments in (induced) pluripotent cells would be an informative next step for the programmed production of urothelial cells for therapy.
