# Supplementary material for: Differential transcription factor expression by human epithelial cells of buccal and urothelial derivation
Source: Exp Cell Res. 2018 Aug 15;369(2):284–94. doi: 10.1016/j.yexcr.2018.05.031 (PMC6092173; doi:10.1016/j.yexcr.2018.05.031)
Supplement: Supplementary file 1 [file mmc1.pdf]

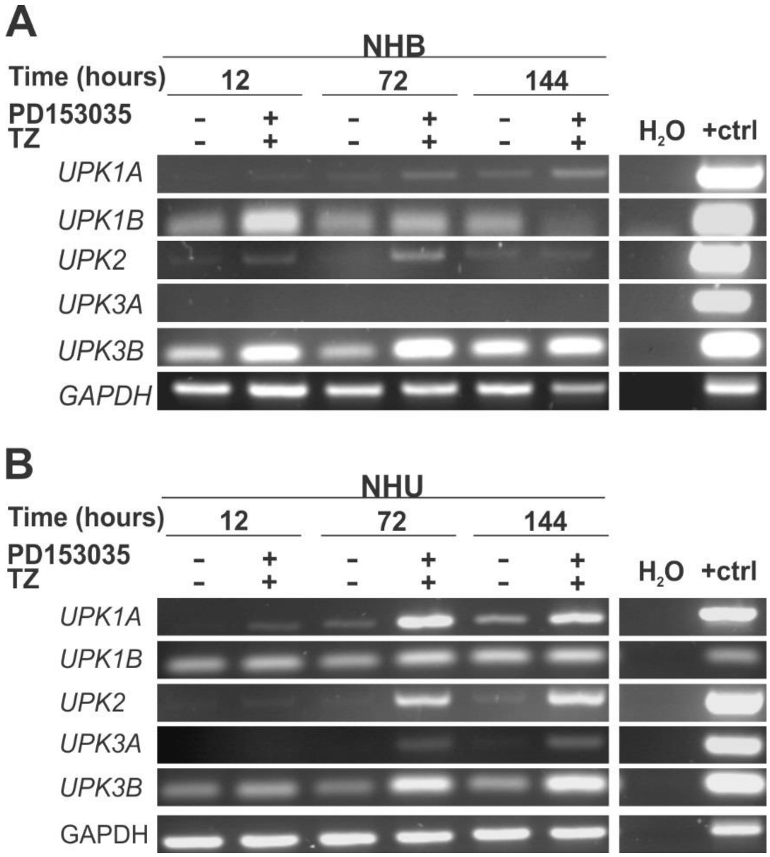

Supplementary Figure 1

Assessment of uroplakin gene expression by NHB and NHU cells *in vitro*.

(A-B) RT-PCR of uroplakin (*UPK1A, UPK1B, UPK2, UPK3A and UPK3B*) mRNA expression by (A) NHB and (B) NHU cell lines on RNA extracted at 12, 72 and 144 hours following treatment with TZ/PD. *GAPDH* was included as a housekeeping gene. RNA was digested with DNAase to remove any genomic DNA contamination and tested as RT-negative controls (not shown). Other shown controls include a no-template (H_2O) negative and a genomic DNA positive control (+ctrl). Experiments were performed on n=2 independent NHB cell lines with similar results.
